# Complete mitochondrial genome of the European smelt *Osmerus eperlanus* (Osmeriformes, Osmeridae)

**DOI:** 10.1080/23802359.2018.1483768

**Published:** 2018-07-10

**Authors:** Evgeniy S. Balakirev, Alexandra Yu Kravchenko, Nikolai S. Romanov, Francisco J. Ayala

**Affiliations:** a Department of Ecology and Evolutionary Biology, University of California, Irvine, CA, USA;; b National Scientific Center of Marine Biology, Far Eastern Branch, Russian Academy of Sciences, Vladivostok, Russia;; c School of Natural Sciences, Far Eastern Federal University, Vladivostok, Russia

**Keywords:** European smelt *Osmerus eperlanus*, *O. mordax*, Osmeridae, Mallotus, Hypomesus, Osmerus, mitochondrial genome

## Abstract

The complete mitochondrial (mt) genome was sequenced in two individuals of the European smelt *Osmerus eperlanus*. The genome sequences are 16,608 and 16,609 bp in size, and the gene arrangement, composition, and size are very similar to the other smelt mt genomes previously published. The difference between the two genomes studied is low, 0.05%. The difference is significantly higher (6.95%) between the *O. eperlanus* genomes studied here and the genome of the congeneric species, *O. mordax* (HM106493.1) available in GenBank. The distribution of divergence is non-uniform along the genomes. There is a continuous segment (≈2.5 kb) encompassing tRNA-Phe, complete 12S rRNA gene, tRNA-Val, and partial 16S rRNA gene, which demonstrates significantly lower levels of divergence than on average for the whole genome. The 12S rRNA and 16S rRNA genes are frequently used for phylogenetic and molecular taxonomy analyses and could underestimate the level of divergence between osmerid fishes.

The European smelt *Osmerus eperlanus* (Linnaeus) has wide distribution in the North Atlantic regions (including estuaries and large coastal lakes) from the southern part of the White Sea to the western coast of France, including the Baltic Sea, the southern part of the North Sea, and the British Islands (McAllister 1984). *O. eperlanus* along with other smelts were previously investigated with a number of short fragments of mitochondrial (mt) and nuclear genes (e.g. Ilves and Taylor 2009; Skurikhina et al. 2013). However, the phylogenetic relationships of osmerid fishes based on short DNA fragments remain unresolved (Ilves and Taylor 2009; Skurikhina et al. 2013 and references therein). To increase the power of phylogenetic analysis of this problematic group of fishes, we have sequenced two complete mt genomes of *O. eperlanus* (GenBank accession numbers MH238073 and MH238074) from the Gulf of Finland of the Baltic Sea (59°56′ 35,3796″ N; 29°37′ 29,085″ E). The primers were designed with the program mitoPrimer_V1 (Yang et al. 2011). The fish specimens are stored at the museum of the National Scientific Center of Marine Biology, Vladivostok, Russia (www.museumimb.ru) under accession numbers MIMB35004 and MIMB35005.

The *O. eperlanus* mt genome sequences are 16,608 and 16,609 bp in size and the gene arrangement, composition, and size are very similar to the smelt fish genomes previously published. We detected eight single-nucleotide and one length differences between the haplotypes OSEP487 and OSEP489; total sequence divergence (*D*_xy_) was 0.0005 ± 0.0001. The comparison of mt genomes now obtained with other complete mt genomes of related groups available in GenBank including genera *Hypomesus*, *Osmerus*, *Mallotus*, *Plecoglossus*, and *Salangichthys* reveals a close affinity of *O. eperlanus* to congeneric species *O. mordax* (HM106493.1) (Figure 1). The difference (*D*_xy_) between them is 0.0695 ± 0.0016, which is in close agreement with the values of interspecific divergence previously reported for the osmerid fishes (e.g. Skurikhina et al. 2013 and references therein).

**Figure 1.**
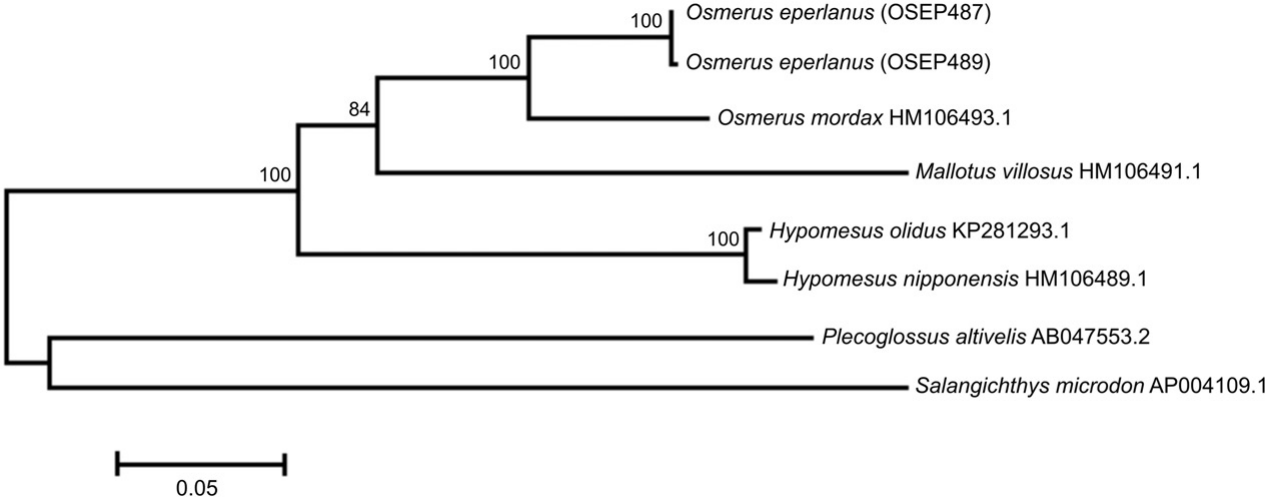
Maximum likelihood tree for the European smelt *Osmerus eperlanus* specimens OSEP487 and OSEP489, and GenBank representatives of the order Osmeriformes. The tree is based on the General Time Reversible + gamma + invariant sites (GTR + G+I) model of nucleotide substitution. The numbers at the nodes are bootstrap percent probability values based on 1000 replications (values below 70% are omitted).

The distribution of divergence between the genomes of *Osmerus eperlanus* (specimens OSEP487 and OSEP489), *O. mordax* (HM106493.1), *Hypomesus olidus* (KP281293.1), *H. nipponensis* (HM106489.1), *Mallotus villosus* (HM106491.1), *Plecoglossus altivelis* (AB047553.2), and *Salangichthys microdon* (AP004109.1) in pairwise comparisons is non-uniform. There is a continuous segment (≈2.5 kb) encompassing the tRNA-Phe, complete 12S rRNA gene, tRNA-Val, and partial 16S rRNA gene, which demonstrates significantly lower levels of divergence than on average for the whole genome. The average nucleotide difference for this low divergence segment is 0.0611 ± 0.0032, but 2.4 times more, 0.1460 ± 0.0018, for the rest of the genome. The 12S rRNA and 16S rRNA genes (main ‘contributors’ to the low divergence segment) are frequently used for phylogenetic and molecular taxonomy analyses (e.g. Ilves and Taylor 2007, 2008, 2009; Chen et al. 2014) and could underestimate the level of divergence between osmerid fishes.
